# Acute Myocarditis Following COVID-19 Vaccination in a Young Adult

**DOI:** 10.7759/cureus.50592

**Published:** 2023-12-15

**Authors:** Parth Vikram Singh, Stephanie A Izquierdo, Sahithya Ekasi, Taylor Schnepp, Claudia Rabanal, Lester Jan A Olimba, Mark Dickinson

**Affiliations:** 1 Department of Research and Academic Affairs, Larkin Community Hospital, South Miami, USA; 2 College of Medicine, St. George's University, St. George's, GRD; 3 College of Medicine, Ross University School of Medicine, Bridgetown, BRB; 4 College of Medicine, University of Medicine and Health Sciences, Camps, KNA

**Keywords:** cardiac troponin, vaccine side effects, covid-19, a case report, myocarditis

## Abstract

The mRNA vaccines for coronavirus disease-2019 (COVID-19) have been implemented across the globe for both emergent and non-emergent applications. We present a rare case of myocarditis following the second dosage of COVID-19 vaccine. In this case, myocarditis was suspected by troponin and erythrocyte sedimentation rate (ESR) levels prior to echocardiography, which demonstrated mild pericardial effusion, mild tricuspid regurgitation, and mild asymmetric left ventricular hypertrophy. Mild to moderate symptoms of myocardial inflammation persisted throughout the patient’s admission, which attributed to the clinical presentation of chest pain and palpitations. As the patient had no relevant history to account for cardiac pathologies prior to vaccination, this case report serves to further investigate the association between mRNA-derived vaccination and subsequent acute myocarditis development.

## Introduction

In clinical trials, three vaccines with Food and Drug Administration approval (PfizerBioNTech, Moderna, and Janssen) showed efficacy and safety without serious side effects [1]. Although, new research raises concerns about the potential link between coronavirus disease-2019 (COVID-19) immunization and severe cardiac events, with unknown pathophysiology [2,3]. In this case report, we intend to present a case of myocarditis linked with COVID-19 vaccination.

## Case presentation

A 25-year-old male presented to the emergency department of Larkin Community Hospital, South Miami with a two-day history of palpitations and chest discomfort. He received multiple vaccines (final dose of COVID mRNA vaccine and single shots of live attenuated influenza vaccine (LAIV); HPV; hepatitis B; and tetanus, diphtheria, and pertussis (TDaP) during his immigration into the USA by the government agencies, three days prior to his symptoms. Following these vaccinations, he experienced abdominal discomfort, vomiting, palpitations, and burning chest pain as well as anxiety that he attributed to these symptoms. 

The patient completed his COVID-19 vaccine series and reported no symptoms or allergic reactions from the past COVID-19 vaccines. He denied any previous cardiac or other medical history. No alcohol or drug use was reported. The patient admits to using electronic tobacco products for the past two months. The patient has no known allergies. There is no pertinent family history of cardiac conditions. He had no recent hospitalizations. The review of symptoms was unremarkable except for palpitations and anxiety. 

On physical examination, the patient was well-developed for his age and a well-nourished man who was awake, alert, and oriented in time, place, and person. The patient was in no acute distress. Cardiovascular examination revealed regular rate and rhythm with a normal S1 and S2. There were no gallops, murmurs, or rubs; a normal point of maximal impulse palpated in the precordium to the left fifth intercostal space, half-inch medial to the left midclavicular line that was normal in character; and no jugular venous distention. The rest of the physical examination was unremarkable. The patient's blood pressure was 133/86 mmHg, pulse was 74/min, respiratory rate was 15, temperature was 98.7°F, and pulse oximetry was at 100%. 

Standard protocol for a patient presenting with chest pain in the inpatient setting includes serum biomarker analysis, cardiac electrical conduction analysis (electrocardiogram (ECG)), and structural evaluations of the heart (echocardiogram). Differentials for a patient presenting with palpitations or chest pain may include acute coronary syndrome, pericardial tamponade, myocarditis, pericarditis, stable/unstable angina, Prinzmetal angina, hypertrophic obstructive cardiomyopathy, congestive heart failure, aortic dissection, and pulmonary embolism. Non-cardiac differentials may include sickle cell crisis (acute chest syndrome), pleural effusion, pancreatitis, peptic ulcer disease, and gastroesophageal reflux disease. 

Investigations performed on this patient specific to his condition included serum troponin and creatine kinase-myocardial band (CK-MB) levels, ECG, echocardiogram, viral serology, and chest X-ray. Myocarditis was suspected based on the patient’s clinical signs and symptoms in combination with acutely increased troponin levels during the episode of palpitations. On laboratory testing, results showed elevated troponin levels (0.76 ng/mL). Our medical team decided to admit the patient to the hospital. Over the course of a day, repeat troponin testing revealed an increase in level (1.60 ng/mL) (the normal range is between 0 and 0.04 ng/mL). Our cardiology service was then consulted. Further testing was ordered by the cardiology service, which included serial ECG, echocardiogram, serial troponin, erythrocyte sedimentation rate (ESR), c-reactive protein (CRP), drug screen, and lipid panel. 

**Figure 1:**
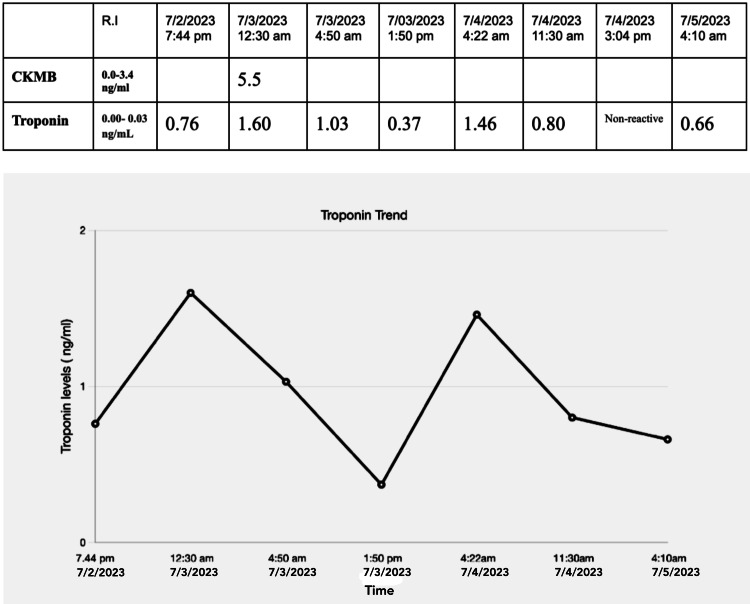
Troponin trends and CK-MB level CK-MB: creatine kinase-myocardial band

**Figure 2:**
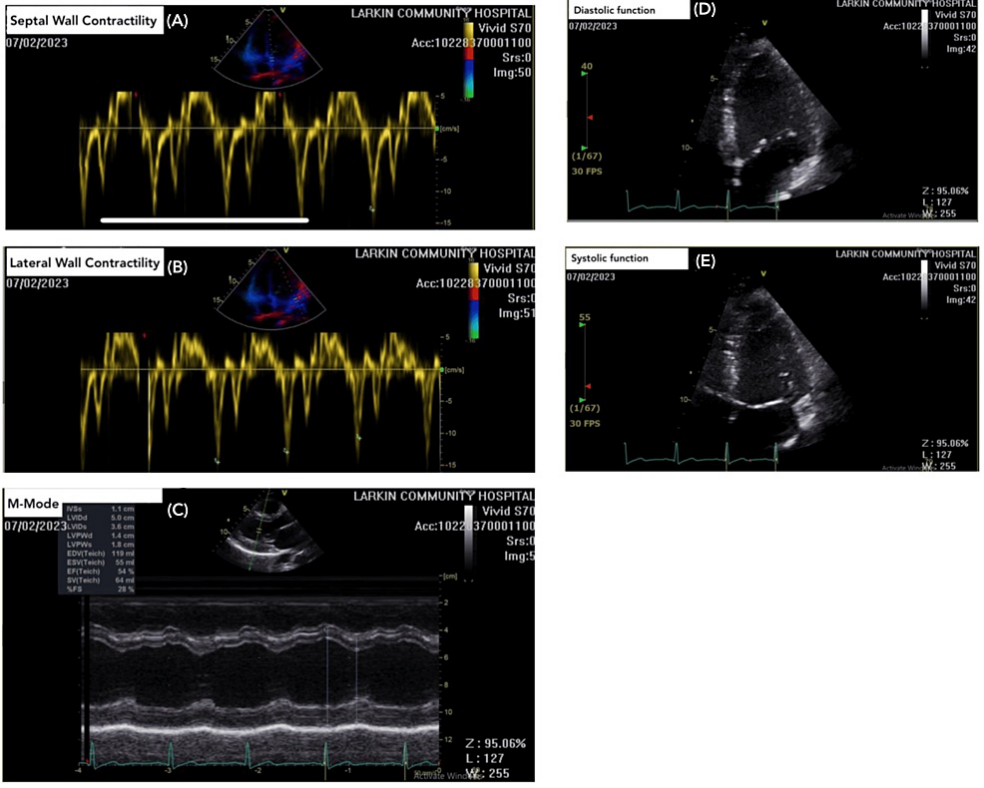
Transthoracic echocardiogram results of a 25-year-old male patient presenting with elevated troponins and complaints of chest pain (A) Septal wall view revealed mild asymmetric left ventricular hypertrophy; (B) Lateral wall view revealed left ventricular filling pattern is normal for age, normal left ventricular systolic function, and normal LVEF of 55%. (C) Motion-mode view shows aortic valve is grossly normal in structure. (D) Diastolic view shows mitral valve and tricuspid valve are grossly normal in structure. Mild mitral regurgitation. (E) Systolic view revealed mild tricuspid regurgitation with an RVSP of 25 mmHg and trace-to-mild pericardial effusion. The aortic root is normal in size. LVEF, left ventricular ejection fraction; RVSP, right ventricular systolic pressure

**Figure 3:**
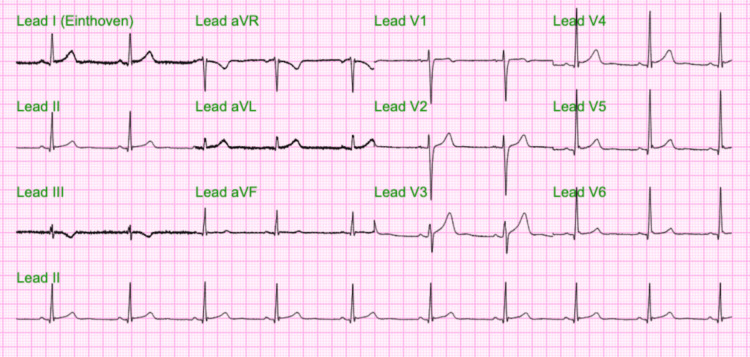
ECG showing minimal ST changes on leads V5-V6 ECG, electrocardiogram

**Figure 4:**
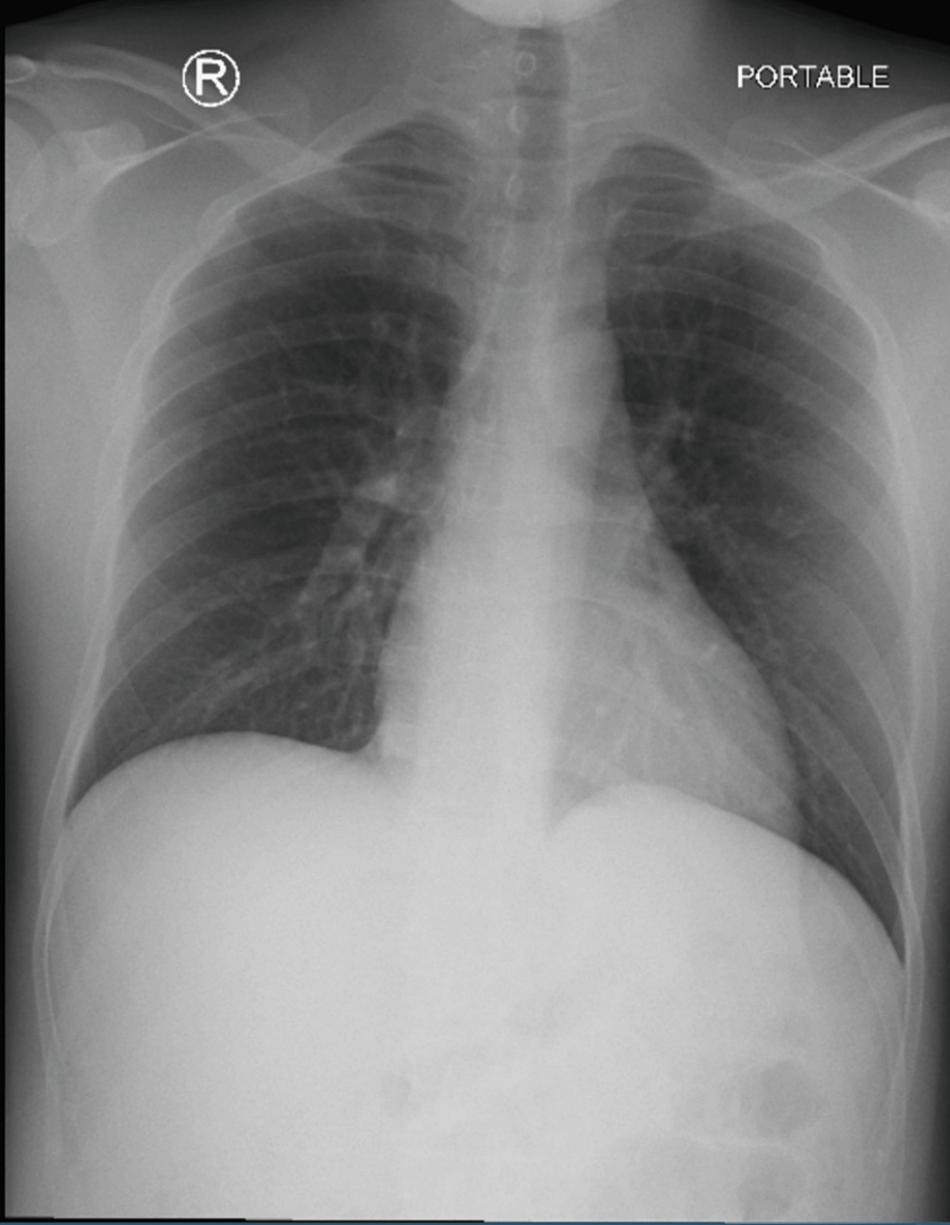
Chest X-ray anterio-posterior view. The cardiomediastinal silhouette is within normal limits in size

Troponin levels fluctuated from July 2nd to July 5th, 2023 ranging between 0.76 and 0.66 (normal level: <0.03 ng/mL) (Figure 1). The patient’s CK-MB level was elevated at 5.5 ng/mL (normal level: 0.0-3.4 ng/mL), as well as the ESR at 18 mm/hr (normal level: 0-15 mm/hr). The echocardiogram showed evidence of race to mild pericardial effusion, mild tricuspid regurgitation, mild asymmetric mild left ventricular hypertrophy, and a left ventricular ejection fraction (LVEF) of 55%. Changes on the echocardiogram were not significant enough to use for the diagnosis of myocarditis solely (Figure 2). Decompensation of the heart after an acute episode of myocarditis may occur such as decreased ejection fraction and diffuse hypokinesis. As a result, a follow-up echocardiogram in a month was recommended to the patient in order to assess for these changes. Viral enzyme-linked immunosorbent assay (ELISA) and polymerase chain reaction (PCR) for COVID-19, hepatitis B, human immunodeficiency virus (HIV), Epstein-Barr virus (EBV), coxsackievirus, influenza virus as well as herpes simplex virus type 1 and 2 were all negative. ECG showed mild ST changes in leads V5-V6 (Figure 3). Anterior-posterior X-ray of the chest was unremarkable (Figure 4). The drug screen was negative for cocaine, THC, barbiturates, benzodiazepines, opiates, and phencyclidine.

Cardiac magnetic resonance imaging (MRI) is indicated but was not performed because of limited facility resources. While endomyocardial biopsy (EMB) is the gold standard for a myocarditis diagnosis, biopsy samples in otherwise healthy patients pose serious risks and complications. EMB would have been considered had the patient been in critical condition or hemodynamically unstable. A pathologic EMB with evidence of myocarditis would have shown inflammatory infiltrates of the myocardium with myocyte necrosis.

Upon review of the results, the service decided to manage this patient as a case of myocarditis. In this case, the lack of a history of viral symptoms or positive viral serologies including ELISA or COVID-19 PCR test led us to assume that the vaccine, rather than an acute infection, was most likely to blame for the myocarditis. These immunization-associated myocarditis are most likely due to the hyperimmunity to vaccines. The cardiovascular service started the patient on losartan 25 mg tablet daily and metoprolol 25 mg tablet every 12 hours. On the fourth day of admission, the patient reported no chest pain, palpitations, and shortness of breath. He was medically stable and cleared for discharge. He was told to refrain from rigorous physical activity for three months and was discharged with beta-blockers (metoprolol 25 mg tablet every 12 hours) with angiotensin-converting enzyme (ACE) inhibitors (losartan 25 mg tablet daily) to help avoid cardiac remodeling. A follow-up visit in the next month is set up for the patient with the cardiologist.

## Discussion

Vaccines, a very important aspect of community medicine, have some inherent risks. Pain, edema, and erythema at the injection site are some of the local side effects. COVID-19 vaccine also has some generalized complications like tiredness, headache, body aches, and pyrexia, among others. However, repeat doses tend to have more severe side effects compared to the initial dose [4].

Myocarditis has historically also been associated with a number of immunizations, with smallpox immunization having the greatest correlation [5]. One of the more severe side effects associated with mRNA vaccines like Pfizer and Moderna is myocarditis, with manifestations extending from pyrexia and slight chest discomfort, dysrhythmias, congestive cardiac failure, and death [4].

Vague clinical constellations, absence of any specific blood marker, and long window period before findings become apparent on echocardiography can make diagnosing myocarditis very challenging regardless of the underlying cause [6,7]. Therefore, the presumptive diagnosis should be made by a skilled physician on the basis of the entire clinical picture like symptoms, increased inflammatory markers like ESR or CRP, cardiac injury biomarkers like cardiac troponin or CK-MB, and imaging [8]. The diagnosis should be confirmed by EMB only if the benefits of diagnosis significantly outweigh the risks associated with the procedure; hence, EMB is usually only indicated in hemodynamically unstable cases with recent onset of reduced ejection fraction [9]. For histopathology, the Dallas criteria were established to aid in the diagnosis of myocarditis, which requires an inflammatory infiltrate and associated myocyte necrosis or damage not characteristic of an ischemic event [9]. Treatment plan in mild cases of myocarditis generally includes exercise avoidance and drugs for the prevention of future complications, like congestive cardiac failure and dysrhythmias. In select cases that also have pericarditis along with myocarditis, non-steroidal anti-inflammatory drugs (NSAIDs) and colchicine are also prescribed [10].

Although viruses are the leading cause of myocarditis, particularly influenza virus and parvovirus B19, in our case the patient showed no signs of recent viral infection like diarrhea, upper respiratory tract infection (URTI), cough, coryza, joint pains, myalgias, fatigue, and fever, which effectively ruled out the viral causes of myocarditis in our patient. The patient showed no clinical signs or symptoms indicating any drug abuse and the drug screen was also negative for cocaine, THC, barbiturates, benzodiazepines, opiates, and phencyclidine ruling out any drug-related toxicity [6].

On physical examination, our patient had normal blood pressure and sinus rhythm, and no signs of heart failure. On initial ECG, very mild ST-segment elevation was noted in lateral leads (V5,V6). Increased cardiac troponin and CK-MB on presentation raised the suspicion of myocardial injury. Serial troponin measurement done, thereafter, showed a further increase in troponin level peaking at 1.6 ng/mL on day two of admission, confirming the element of myocardial injury. Subsequently, a transthoracic echocardiogram was conducted, which showed trace pericardial effusion, mild tricuspid regurgitation, and mild asymmetric left ventricular hypertrophy but no wall motion abnormality was detected and LVEF was found to be normal. Due to normal LVEF and the absence of any wall motion abnormalities, it was decided to avoid EMB since it would not have any consequence on the treatment plan for the patient. Medical treatment including losartan and metoprolol was started for protection against complications of myocarditis including heart failure and arrhythmia, respectively. The patient’s symptoms subsided and he showed decreasing trends of troponin and other biomarker after day two of admission. The patient was discharged on the fourth day of admission with a recommendation to avoid exercise, continue the medicine for a month, and follow up with cardiology after one month.

According to the Ministry of Health, Israel, men under 30 had a greater incidence of myocarditis; this compares to a rate of 1/100,000 for the entire population and 1/20,00 for people between the ages of 16 and 30, with 62 incidences of acute myocarditis out of the nearly five million people who received the COVID-19 vaccine and of the 62 patients, two passed away. Only 56 cases were documented after the repeat dosage of the COVID-19 vaccine, while only the remaining six cases were after the first dose [5]. 

Furthermore, the U.S. Department of Defense noted that out of the 2.7 million military servicemen who had received the COVID-19 vaccine, 14 had developed myocarditis, that is an incidence of 0.52/100 000 and out of 14, 11 had obtained the Moderna vaccine, and three of the personnel received Pfizer vaccine. With 13 of them after their repeat dose of COVID-19 vaccines and only one after the first COVID-19 shot [6]. There fails to be a lot of data available on the process because myocarditis cases following COVID-19 vaccination are quite rare. There have been multiple speculated rationales, nevertheless, for the disease's recurrence after vaccination. The three main ways COVID-19 mRNA vaccines can trigger hyperimmunity are autoimmune reactions to the vaccine's mRNA, cross-reactivity between antibodies to severe acute respiratory syndrome coronavirus 2 (SARS-CoV-2) spike glycoproteins and myocardial contractile proteins, as well as differences in the signaling of hormones between men and women. Immunogenetic background, age, and sex can all have an influence on these processes [7].

The immune system might interpret the vaccine's mRNA for an antigen, igniting immunological pathways and pro-inflammatory cascades in the heart. One of the postulated causes could be the molecular mimicry between cardiac self-antigens and the spike protein of SARS-CoV-2. The myocardial-myosin heavy chain represents a human protein sequence, which could cross-react with antibodies linked to SARS-CoV-2 spike glycoproteins. These autoantibodies could just be repercussions of inflammation of the myocardium and injury, or they could be an indicator of an immune-genetic background that contributes to hyperimmunity leading to myocarditis as a reaction to a trigger [11].

The development of the myocarditis brought on by the COVID-19 mRNA vaccine may be controlled by variations in hormone signaling. Given that male patients have a higher incidence compared to female patients, testosterone may inhibit anti-inflammatory immune cells while encouraging a powerful T helper 1 cell-type immune-mediated response [4]. On the other hand, estrogen antagonizes pro-inflammatory T cells, which reduces the degree of cell-mediated immune responses [11].

In summary, we can say that out of all the advances in the previous century, vaccination is one of the greatest advances in medicine and is responsible for significantly reducing the incidence of infectious diseases but rarely can also be associated with significant side effects like myocarditis [12].

## Conclusions

We discussed a case of myocarditis most probably after a COVID-19 mRNA vaccine series. After thorough consideration of other probable causes of elevated troponins, elevated ESR levels, and cardiac imaging, we have presumptive evidence of vaccine-related myocarditis most probably from the COVID-19 mRNA vaccine. Though an endomyocardial biopsy is considered the gold standard for diagnosing myocarditis, given the high procedural risk for a benign presentation, the team has decided to forego that and considered clinical evidence for the diagnosis of myocarditis.
